# Decellularized Porcine Cartilage Scaffold; Validation of Decellularization and Evaluation of Biomarkers of Chondrogenesis

**DOI:** 10.3390/ijms22126241

**Published:** 2021-06-09

**Authors:** Roxanne N. Stone, Stephanie M. Frahs, Makenna J. Hardy, Akina Fujimoto, Xinzhu Pu, Cynthia Keller-Peck, Julia Thom Oxford

**Affiliations:** 1 Interdisciplinary Studies Program, Boise State University, Boise, ID 83725, USA; roxannestone@u.boisestate.edu; 2 Biomolecular Research Center, Boise State University, Boise, ID 83725, USA; StephanieTuft@boisestate.edu (S.M.F.); makennahardy@u.boisestate.edu (M.J.H.); akinafujimoto@boisestate.edu (A.F.); shinpu@boisestate.edu (X.P.); ckpeck@boisestate.edu (C.K.-P.); 3 Center of Biomedical Research Excellence in Matrix Biology, Boise State University, Boise, ID 83725, USA; 4 Biomolecular Sciences Graduate Programs, Boise State University, Boise, ID 83725, USA; 5 Department of Biological Sciences, Boise State University, Boise, ID 83725, USA

**Keywords:** cartilage, chondrocytes, decellularized, scaffold, proteomics, real time quantitative PCR, histology, scanning electron microscopy, C28/I2 cells, porcine auricular cartilage

## Abstract

Osteoarthritis is a major concern in the United States and worldwide. Current non-surgical and surgical approaches alleviate pain but show little evidence of cartilage restoration. Cell-based treatments may hold promise for the regeneration of hyaline cartilage-like tissue at the site of injury or wear. Cell–cell and cell–matrix interactions have been shown to drive cell differentiation pathways. Biomaterials for clinically relevant applications can be generated from decellularized porcine auricular cartilage. This material may represent a suitable scaffold on which to seed and grow chondrocytes to create new cartilage. In this study, we used decellularization techniques to create an extracellular matrix scaffold that supports chondrocyte cell attachment and growth in tissue culture conditions. Results presented here evaluate the decellularization process histologically and molecularly. We identified new and novel biomarker profiles that may aid future cartilage decellularization efforts. Additionally, the resulting scaffold was characterized using scanning electron microscopy, fluorescence microscopy, and proteomics. Cellular response to the decellularized scaffold was evaluated by quantitative real-time PCR for gene expression analysis.

## 1. Introduction

Osteoarthritis (OA) is defined as a chronic, debilitating, and painful disease. It is estimated to be one of the leading causes of disability worldwide [1,2,3,4]. Sports, recreational activities, and even daily movements can contribute to the formation of cartilage lesions. Lesions or chondral defects, when left untreated, can lead to degenerative joint disease that may include an inflammatory response [4,5].

Knee OA is the most common type of OA and accounts for 70% of arthritis-related hospital admissions and 23% of clinical visits [2]. Given the anatomical position of the knee, it acts as a shock absorber by withstanding both tension and compression [6]. Hyaline cartilage within articular joints is located at the ends of long bones. Cartilage lacks nerve fibers and is avascular. It is tough, but flexible, and contains large amounts of glycosaminoglycans (GAGs) such as chondroitin sulfate and hyaluronic acid (HA), which interact with type II/IX/XI heterotypic collagen fibrils. Proteoglycans, such as aggrecan, are predominant molecular constituents of articular cartilage [2,4,7].

The precisely organized architecture of the extracellular matrix (ECM) provides the normal structural integrity of tissues. The function of the articular cartilage is to protect the subchondral bone from mechanical forces by distributing the load equally while maintaining low friction across the joint surfaces [2,4,7]. While normal healthy cartilage supports tissue homeostasis and chondrocyte function, osteoarthritic cartilage does not effectively carry out the functions of maintaining cartilage homeostasis and cellular differentiation [4].

Chondrocytes are the predominant cell type in growing cartilage. These cells produce new ECM until the skeleton stops growing at adolescence. Mature chondrocytes rarely divide and have limited ability to proliferate. Chondrocytes have been shown to decrease with age, which may explain, in part, why cartilage lesions do not spontaneously heal [4,6,8]. Older adults, aged 50 and older, are at an increased risk for knee OA and this may be due in part to hormonal changes that are associated with biological aging in the chondrocytes [2]. Osteoarthritic cartilage does not effectively carry out the functions of normal healthy cartilage and treatment options are limited [2,4]. Articular cartilage degeneration begins at the surface and leads to the onset of fibrillation, which disrupts the molecular framework of the ECM [9,10]. These changes may be triggered by mechanical damage or wear and tear of the tissue [11]. The collagen fibrils disorient beneath the surface and a decrease in HA and aggrecan have been reported [12,13]. A better understanding of the cartilage degeneration and regeneration mechanisms would be useful to help develop new potential treatment strategies to repair damaged cartilage. Currently, the primary treatment option for knee OA is full knee replacement [2].

Cell-based strategies provide an alternative to full knee replacement; however, the major limitation to current recellularization approaches through cell therapy is that the outcome is often the formation of fibrocartilage rather than the desired hyaline cartilage [14,15,16]. Additionally, cell retention within the target area is a challenge, and in some cases, cells move to other parts of the body [2,6,8]. A suitable scaffold may alleviate this problem. Current scaffolds used for cartilage regeneration include synthetic and natural materials. Natural materials include agarose, alginate, chitosan, collagen, fibrin, and hyaluronan. Synthetic polymers include polylactic acid (PLA), polyglycolic acid (PGA), and their copolymer polylactic-co-glycolic acid (PLGA). Scaffolds used in tissue engineering approaches for cartilage regeneration have recently been reviewed in Huang et al. [17]. Hybrid approaches have been used in which cartilage insets are placed within a 3D-printed scaffold [18], showing promising results histologically. Biomaterials designed with improved cell adhesion that can promote differentiation leading to healing in the damaged tissue can supplement cell-based approaches for the treatment of cartilage lesions and OA treatment [2,4,5].

In this study, decellularization strategies for cartilage were evaluated based on their ability to remove DNA and other cellular material while preserving extracellular matrix components of the original tissue. We performed decellularization using a combination of chemical and physical methods. Surfactants, acid and bases, and enzymes were included in the chemical and enzymatic treatment to remove cells [19,20,21]. Following decellularization, C28/I2 human chondrocytes, which were established by transfection of primary cultures of juvenile costal chondrocytes [22], were seeded onto the scaffold and the cellular response to the scaffold was evaluated.

## 2. Results

**Figure 1:**
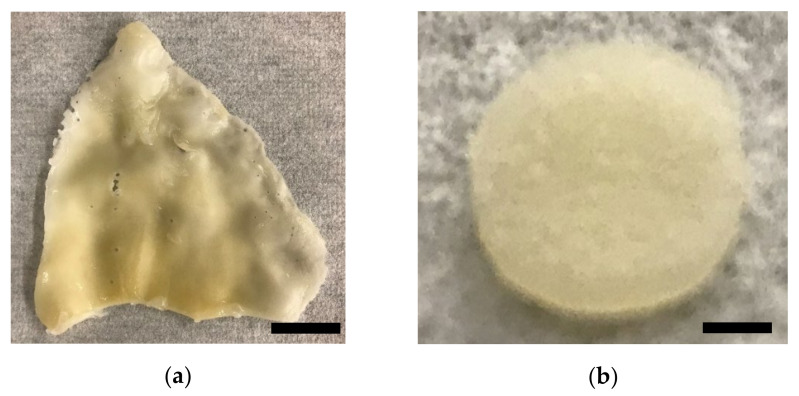
Cartilage scaffold resulting from decellularization process: (**a**) porcine auricular cartilage after dissection and initial processing; (**b**) 8 mm diameter cartilage disc after decellularization process was complete. Discs of decellularized cartilage were formed using an 8 mm diameter biopsy punch. Scale bars: (**a**) 30 mm; (**b**) 2 mm.

After removal of all tissue from the surface of the cartilage, the cartilage tissue was revealed as shown in Figure 1a. Discs of 8 mm diameter and 1.08 mm thickness were created from the larger cartilage (Figure 1b).

**Figure 2:**
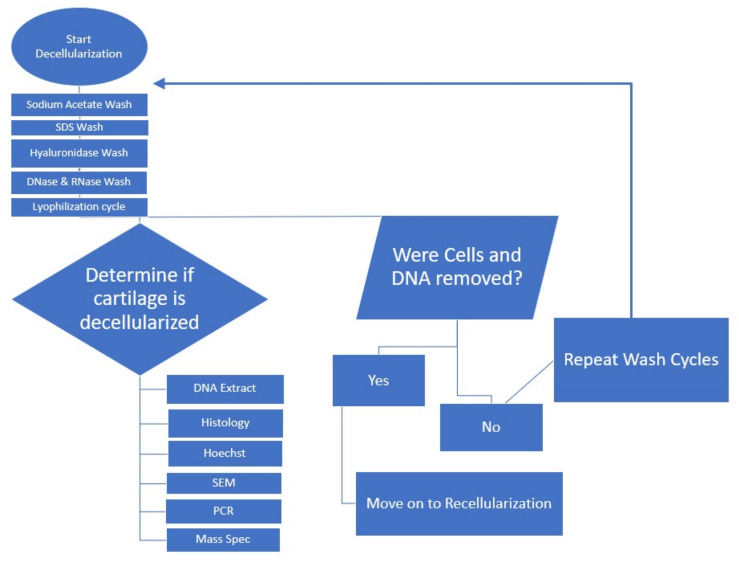
Flowchart for decellularization process.

**Figure 3:**
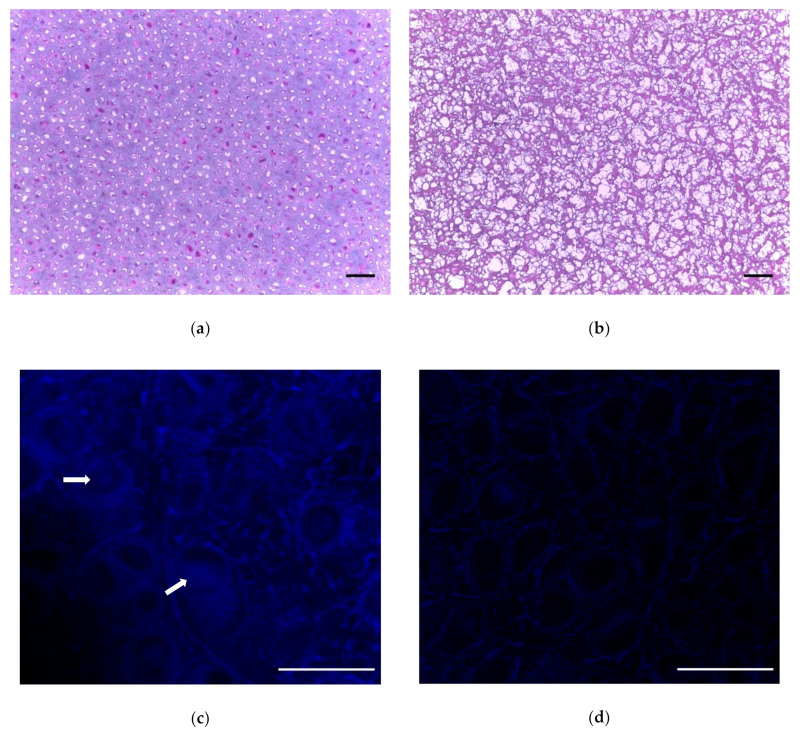
Visualization of cartilage tissue by histology. (**a**) Nondecellularized porcine cartilage stained with H&E, 10× transverse image. (**b**) H&E stain on final decellularized cartilage, transverse image. (**c**) Hoechst stain to fluorescently visualize DNA (blue). Arrows indicate nuclei. (**d**) Hoechst stain shows absence of DNA after decellularization process (see absence of blue). Scale bar = 100 µm for (**a**,**b**); scale bar = 50 µm for (**c**,**d**).

After a subsequent 24 h hyaluronidase treatment [23], followed by a 24 h 37 °C DNase and RNase treatment [19,23], we evaluated the tissues by histology and scanning electron microscopy. Freeze–thaw cycles were repeated followed by DI water incubation, and SDS treatment at 37 °C, with subsequent DNase digestion for 72 h at 37 °C with agitation. The overall decellularization process is depicted in Figure 2. Results from histological analysis are shown in Figure 3a,b. Histological analysis showed a decrease in cellular structures with the preservation of the extracellular matrix. Hoechst staining showed DNA content to be reduced (Figure 3c,d).

**Figure 4:**
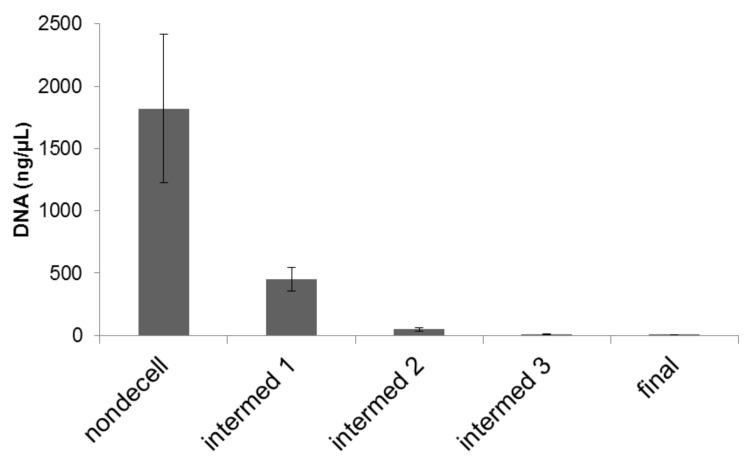
DNA content before, during sequential stages, and after decellularization process. DNA was extracted and quantified spectrophotometrically. Quantitative measurements of DNA within scaffolds before, during, and after decellularization process indicated that residual DNA was at approximately 24.76%, 2.63%, 0.66%, and finally 0.22% of the original content. Error bars: Mean ± standard error of the mean. N = 6.

To quantify the depletion of DNA in the samples, samples were subjected to a DNA extraction process before and after decellularization. We found that the decellularization process removed 98.8% of the DNA associated with cells (Figure 4).

**Figure 5:**
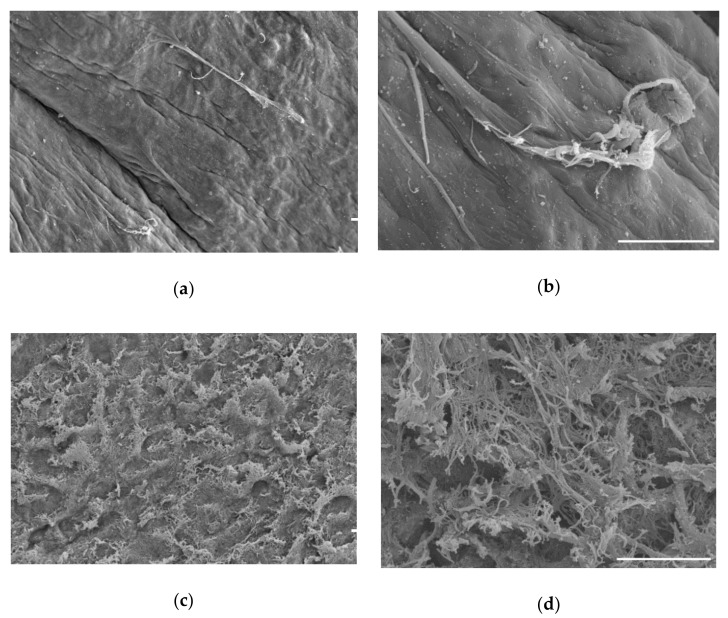
Visualization of cartilage by scanning electron microscopy. (**a**) Cartilage prior to treatment; (**b**) higher magnification of cartilage prior to treatment, where the tissue appears smooth and intact; (**c**) final decellularized cartilage disc; (**d**) higher magnification of decellularized cartilage disc. Appearance after decellularization indicates a rough surface with increased surface area and exposed collagen fibrillar networks. Scale bars = 20 µm.

Scanning electron microscopy was performed to monitor tissue throughout the decellularization process. Surface features are visualized in Figure 5. Prior to the decellularization process, cells, although present within the tissue, are not visible at the surface of the cartilage samples at either low (Figure 5a) or high magnification (Figure 5b). The surface features revealed by the decellularization process and scanning electron microscopy demonstrate an increase in surface complexity and exposure of the collagenous fibrillar matrix existing within the tissue (Figure 5c,d).

To complement the analysis of DNA depletion after treatment, we carried out mass spectrometry to analyze the protein content of the scaffold before and after treatment for decellularization. Nuclear proteins were considered for their potential as suitable biomarkers for decellularization. We investigated a profile of 20 nuclear proteins that included APTX, UIMC1, DMRT1, H3F3A, MX1, ISG20, IREB2, MYOCD, NFATC1, NROB1, PTBP1, POU5F, SORBS2, SRPK3, SREBF1, HISTONE H4, STAT5A, HNF1B, DHX16, and RAG1. Of these, 15 were depleted to a level that rendered them no longer detectable by mass spectrometry. Five of these (HISTONE H4, STAT5A, HNF1B, DHX16, and RAG1) were depleted by 83–96%, indicating that there may be biochemical interactions mediating molecular interactions independent of the cellular compartment. We propose a nuclear protein profile of 15 proteins that may be used to assess and evaluate the efficiency of the decellularization process. The nuclear proteins comprising this profile are listed in Table 1.

In addition to nuclear biomarkers, we also analyzed organellar cellular proteins including those associated with mitochondria, Golgi, and endoplasmic reticulum. We investigated five Golgi-specific proteins, 29 mitochondrial proteins, and 20 endoplasmic reticulum proteins that were depleted as a result of the decellularization process. We found that the Golgi proteins B3GALNT1, MAN1A1, FUT2, and MGAT4C represented a protein profile that may be suitable to monitor cellular depletion during decellularization processes, however the Golgi protein B3GNT5 was not fully depleted in our experiments potentially due to secondary interactions. Golgi proteins and the extent to which depletion was observed are listed in Table 2.

We investigated mitochondrial proteins to determine a protein profile of mitochondrial biomarkers that may serve as a reference set to provide more reliable indicators of decellularization. We measured the protein content of 29 mitochondrial proteins and found that 19 of these (ACO2, AKAP10, GOT2, CPT1B, CYP11A1, ATC4D, CYBB, COX17, CYP11B1, GPAM, GATM, MUT, RHOT2, UCP2, UCP3, MT-ND5, SDHA, CUCLG1, and VARS2) were efficiently depleted through the decellularization process. In contrast, some mitochondrial proteins were detected after the decellularization process, indicating that they may not be reliable indicators of decellularization. Mitochondrial proteins and their extent of depletion are listed in Table 3.

We investigated endoplasmic reticulum proteins by mass spectrometry before and after decellularization and found that a protein profile comprising 16 prevalent endoplasmic reticulum proteins may be considered as reliable biomarkers for decellularization processes. These include RPS13, RPS3, CYP8B1, RPL14, RPL6, CRYBB1, RPN2, HSPA5, FOLH1, HSPA1A, HSPA1B, HSPA1L, HMOX1, GANAB, ATP2A2, and VCP. Other endoplasmic reticulum proteins detected by mass spectrometry were not as efficiently removed from the tissue by the decellularization process and may indicate non-specific interactions that would render these proteins unreliable as indicators of decellularization. Endoplasmic reticulum proteins and the extent to which they were depleted during the decellularization process are listed in Appendix A, Table A1.

We investigated 65 membrane proteins to analyze the extent to which they were depleted during the decellularization process and found 38 that were reliably serve as indicators of decellularization. The biomarker profile of membrane proteins include ARF6, ALOX15, CAPN1, CXCR4, CYSLTR2, DSG1, EDNRA, GJA1, RABGGTA, GGT1, GHR, GNAQ, ITGB1, IFNAR1, IL4R, IL6R, LDLR, KIT, STEAP1, NTRK3, PTH1R, PDZD11, ATP2B1, PECAM1, PCDH11X, RAMP1, SAG, SIGLEC1, SLA-DQCA, SLA-DQCB, SLA-DQDB, KCNN3, SLC5A1, SLC22A6, SLC22A7, TPO, TLR9, and TGFBR3. Membrane protein biomarkers that may serve as suitable indicators of cellular depletion are listed in Appendix A, Table A2.

Analysis of cytosolic proteins showed that decellularization depleted the samples of these proteins as well. As shown in Appendix A, Table A3, we identified 40 cytosolic proteins that were depleted to a level exceeding 78%. These profiles may be useful to others that desire to track the efficiency of the decellularization process.

**Figure 6:**
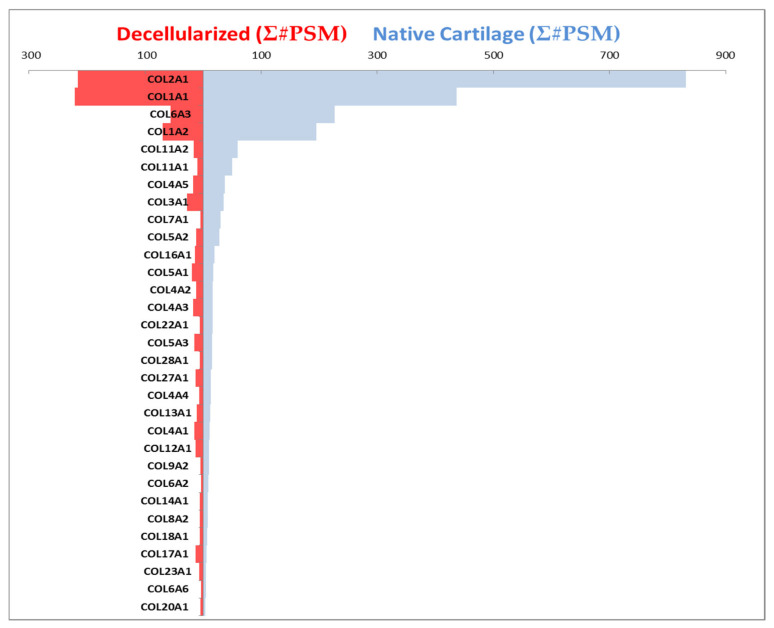
Collagenous composition of the decellularized scaffold. Blue bars show the collagens present in cartilage prior to the decellularization process, and red bars show composition of the scaffold after the decellularization process. The length of the bars to the left and right corresponds to abundance of the specific collagens. Collagen alpha chains are listed in the order of decreasing prevalence within native cartilage. The resulting decellularized scaffold (red) contained COL2A1, COL1A1, COL6A3, COL1A2, COL11A2, COL11A1, COL4A5, COL3A1, COL5A2, COL16A1, COL5A1, COL4A2, COL4A3, COL5A3, COL27A1, COL13A1, COL4A1, COL12A1, and COL17A1. Minor contributions of COL7A1, COL22A1, COL28A1, COL4A4, COL9A2, COL6A2, COL14A1, COL8A2, COL18A1, COL23A1, COL6A6, and COL20A1 were detected after decellularization. Horizontal axes values represent the sum of peptide spectrum matches (PSM), the total number of identified peptides for each collagen as an indication of quantity.

We utilized mass spectrometry to characterize the composition of the scaffold resulting from our process. We quantified extracellular matrix proteins before and after decellularization. As expected, we found that many of the noncollagenous extracellular matrix proteins were significantly depleted during the process, as shown in Table 4. However, collagens were retained within the scaffold, as shown in Figure 6.

Collagen alpha chains were analyzed before and after decellularization to assess collagenous composition of the resulting decellularized scaffold. The collagen alpha chains were detected in native cartilage by mass spectrometry prior to decellularization. After the decellularization process, nine of these were no longer detectable as shown in Figure 6. Blue horizontal bars indicate the composition of cartilage before the decellularization process, and red bars show the composition of the scaffold after the decellularization process. The resulting decellularized scaffold (red) contained COL2A1, COL1A1, COL6A3, COL1A2, COL11A2, COL11A1, COL4A5, COL3A1, COL5A2, COL16A1, COL5A1, COL4A2, COL4A3, COL5A3, COL27A1, COL13A1, COL4A1, COL12A1, and COL17A1. Minor contributions by COL7A1, COL22A1, COL28A1, COL4A4, COL9A2, COL6A2, COL14A1, COL8A2, COL18A1, COL23A1, COL6A6, and COL20A1 were detected in the final decellularized cartilage scaffold (Figure 6). The final decellularized cartilage scaffold did not contain detectable levels of COL5A1, COL10A1, COL8A1, COL21A1, COL25A1, COL9A1, COL6A5, COL15A1, and COL26A1.

**Figure 7:**
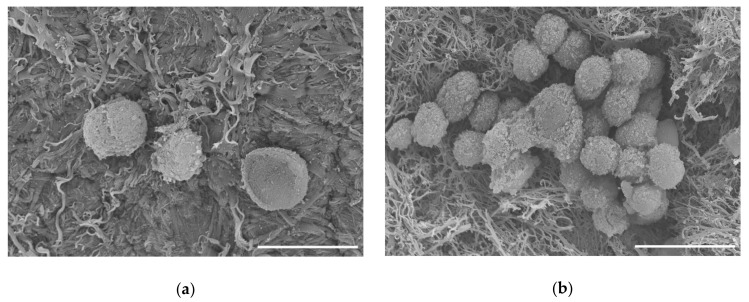
Scanning electron micrographs of C28/I2 chondrocyte cells on decellularized scaffold. (**a**) Cells attached to scaffold after 1 week in culture. (**b**) Cells on scaffold after 8 months in culture. Scale bar = 20 µm.

To test the biocompatibility of our scaffold, we seeded C28/I2 human chondrocyte cells onto the scaffold and maintained these constructs in culture. After one week, we observed cells associated with the surface of our scaffold, as shown in Figure 7. Scanning electron micrographs showed that the cells were maintained on the surface of the scaffold. Cellular clusters were observed after 8 months as shown in Figure 7b.

**Figure 8:**
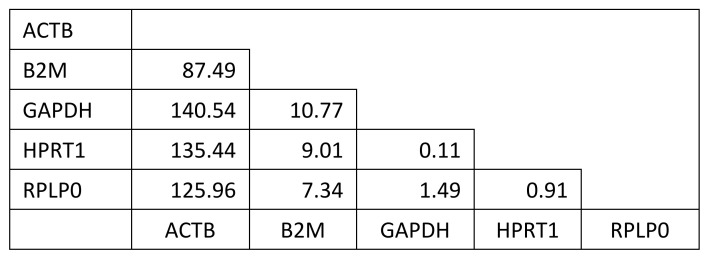
Analysis of variance of difference to determine most suitable housekeeping genes. Candidate housekeeping genes were considered in a pairwise fashion to determine which ones were the most consistently expressed independent of experimental condition within this study. GAPDH and HPRT1 displayed the minimum variance of difference over all experimental conditions and timepoints.

Cellular response was evaluated using quantitative real-time PCR to monitor the expression of genes related to chondrogenic differentiation. Five candidate housekeeping genes were compared for all experimental conditions used in this study to identify those that remain constant and may therefore serve as appropriate housekeeping genes. GAPDH and HPRT1 were selected as the housekeeping gene for normalization in these experiments based on comparison to three other candidate housekeeping genes and were found to be stably expressed independent of experimental conditions based on minimal variance as shown in Figure 8.

**Figure 9:**
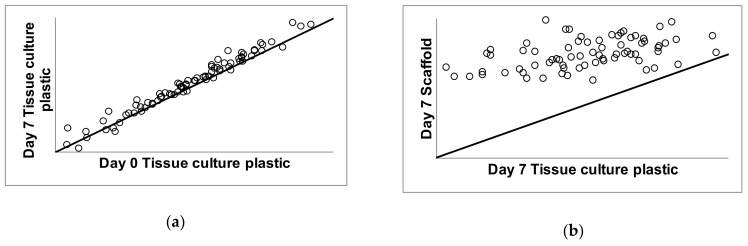
Correlation analysis of gene expression during growth on tissue culture plastic and cartilage scaffold. C28/I2 cells were grown under conventional conditions on tissue culture plastic for one week (**a**) and compared to cells grown on cartilage scaffold for 7 days (**b**). The diagonal line in (**a**,**b**) indicates the trend expected if there was no change between conditions; day 0 versus day 7 on tissue culture plastic in (**a**) and day 7 on tissue culture plastic versus day 7 on cartilage scaffold in (**b**). Data points above the line reflect genes expressed at higher levels on the cartilage scaffold compared to plastic. Data points below the line indicate genes that were expressed at higher levels on plastic compared to the cartilage scaffold. Data points that fall on the line were not changed.

The expression levels of genes related to chondrogenic differentiation were determined relative to housekeeping genes and these relative abundance values were reported as mean plus/minus standard deviation. Correlation analysis of gene expression was carried out to compare gene expression over time on conventional tissue culture plastic (Figure 9a) comparing day 7 to day 0 in culture. Correlation analysis was also carried out to compare gene expression of cells grown on the scaffold to the gene expression levels on tissue culture plastic after 7 days in culture (Figure 9b). The diagonal line indicates the trend expected if there is no change between the cartilage scaffold and plastic. Data points above the line reflect genes expressed at higher levels on the cartilage scaffold compared to plastic (Figure 9b). Data points below the diagonal line indicate genes that were expressed at higher levels on tissue culture plastic compared to the cartilage scaffold. Data points that fall on the line were not changed based on culture conditions.

**Figure 10:**
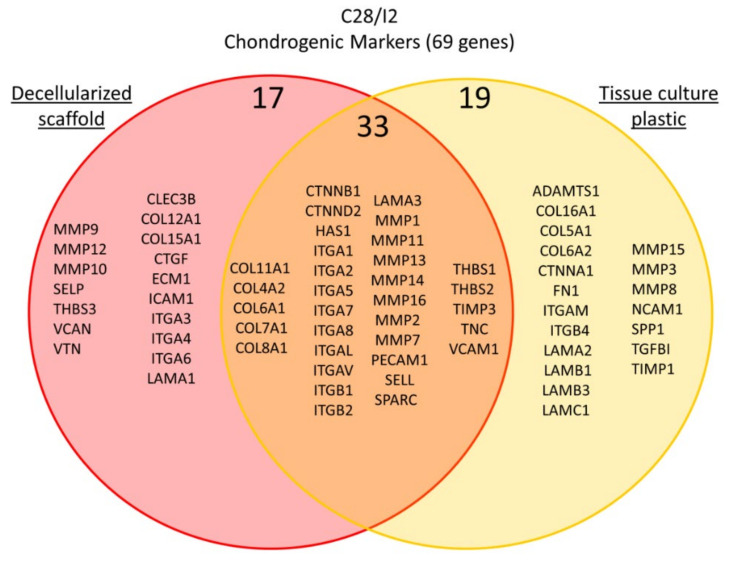
Gene expression of markers in C28/I2 cells. Gene expression for extracellular matrix, cell adhesion molecules, and cell–cell attachment proteins was measured by quantitative real-time PCR. The venn diagram clusters genes with respect to upregulation in cells grown on conventional tissue culture plastic shown in yellow, in cells grown on the decellularized cartilage scaffold shown in pink. Genes that were upregulated under both conditions are shown in the orange overlap region of the Venn diagram.

A total of 77 genes were analyzed during C28/I2 chondrocyte cell culture and differentiation for 7 days. We identified 52 genes that were upregulated from day 0 to day 7 on standard tissue culture plastic and therefore serve as indicators of chondrocyte phenotype. Of those 52 genes, 33 genes also showed an increase in expression when cells were cultured on decellularized 3D porcine scaffold. A total of 19 genes were upregulated in cells maintained on tissue culture plastics but were not upregulated significantly during the same time period by cells cultured on decellularized porcine cartilage scaffold. A total of 25 genes were upregulated in cells grown on decellularized scaffold that were not observed to be upregulated on tissue culture plastic under standard 2D culture conditions and these are summarized in Table 5 and Figure 10.

## 3. Discussion and Conclusions

In this study, we used decellularization to create an extracellular matrix scaffold that supports chondrocyte cell attachment and growth. We evaluated the decellularization process histologically and molecularly. Our conclusions identify new and novel biomarker profiles that may aid future cartilage decellularization efforts. The resulting scaffold was characterized using scanning electron microscopy, fluorescence microscopy, and proteomics. Cellular response to the decellularized scaffold was evaluated by quantitative real-time PCR and mass spectrometry for gene expression and proteomic analysis to analyze collagen content. Our approach demonstrated effective decellularization of a porcine cartilage scaffold by monitoring DNA content before and after decellularization.

Osteoarthritis is one of the leading causes of disability worldwide [1,2,3,4]. Many studies have investigated the use of various scaffolds as provisional chondro-inductive matrices [1,2,5]. In addition to biocompatibility, criteria for tissue engineering composites include (1) resorbability, (2) the ability to resist mechanical stresses, and (3) clinical relevance. Scaffolds must support cell differentiation and maintenance of a mature cartilage phenotype. However, there is no standard decellularization method to date. Previously published approaches include chemical, physical, or combinative methods [6,7,8,19].

The ultimate goal of decellularization is to remove all native genetic information and cellular components from the ECM. Surfactants, acid and bases, and enzymes make up the chemical and enzymatic portion of the process. Mechanical agents are also under study to determine the effectiveness for decellularization of a tissue or organ. These agents typically work by lysing cells through disrupting the phospholipid bilayer of the cell membrane. Ionic surfactants are widely used to remove cells and genetic material [19,20]. Treatments should be applied with continuous shaking [75,76]. Sodium dodecyl sulfate (SDS) currently meets the standard requirements of complete cell removal and elimination of at least 90% DNA [23]. SDS has been shown to damage structural properties if used at high concentration for long durations [6,7,20,21,23]. A comparison of five different decellularization treatments showed that several methods resulted in a significant reduction of DNA. Treatment with 2% SDS for eight hours resulted in the greatest decrease of DNA; with only minor decreased collagen content [6,7,20].

Some conflicting information exists in the literature regarding the duration of 1% SDS washes. The time to reach desired decellularization results range from 24 h to seven days [23,75,77]. The reported results indicate that the number of cells could be significantly reduced from engineered constructs. Higher or lower levels of DNA are most likely related to the thickness of tissue and the concentration and duration of specific detergents [20,23,77].

Only a few studies have explored decellularization of whole cartilage scaffolds for joint regeneration [23,75]. In this study, we used ethanol to defat samples and guanidine hydrochloride and sodium acetate to denature and remove noncollagenous components from cartilage dissected from the ear. Guanidine hydrochloride and sodium acetate have been shown to be effective for denature and remove noncollagenous components [75].

In this study, sodium hydroxide (NaOH) was used to inactivate cellular proteins and pathogens and denature DNA and RNA. Previous studies have shown that NaOH is an effective means of inactivating cellular proteins and pathogens and denaturating DNA and RNA. NaOH treatment removes cells and helps increase the porosity of the tissue [23,75].

Our study included the use of freeze–thaw cycles to help increase the porosity by forming more pores Freeze–thaw cycles have been shown to result in the formation of pores after ice crystal formation in addition to contributing to the disruption of resident chondrocytes. These cycles are often conducted in phosphate-buffered saline (PBS) solution to maintain physiological pH and osmolality, which additionally helps remove the residual reagents [7,19,20,23,76,77,78,79].

In this study, DNase and RNase treatments were used to remove DNA and RNA. DNase and RNase may require as many as three cycles to accomplish complete depletion of native genetic material [7,19,20,23,76,77,78,79]. Removal of 99% of genomic material was observed after a six-day wash cycle in our studies [23]. To quantify the DNA present in cartilage samples before and after decellularization, we used a DNA extraction process [21,79]. The DNA content of the sample was also assessed using Hoechst stain [21,23]. We concluded that the decellularization wash cycles successfully resulted in decellularizing the porcine cartilage scaffold. Traditionally, laboratories have monitored DNA content to determine if cells have been removed since all cells contain DNA. However, DNA may be present even when cells no longer exist due to potential interaction between the matrix and the DNA. Therefore, mass spectrometry was used to measure the removal of cellular proteins including those from the nucleus, mitochondria and Golgi, cytosol, rough endoplasmic reticulum, and plasma membrane. These additional measures more thoroughly demonstrated that the cells were removed at least to a level below the threshold of detection. In this study, lyophilization was used to help with cell disruption and removal of cellular components [78,79]. All samples were sterilized before using for cell culture.

To our knowledge, this study represents the first demonstration of biomarker analysis for decellularization of porcine ear for cartilage regeneration. Additionally, PCR was used to quantify mRNA expression of key chondrogenic differentiation markers expressed by cells after seeding on the scaffold. Chondrogenic marker genes were analyzed for significant changes between control cells grown on tissue culture plastic and those grown under conditions provided by the decellularized scaffold [76]. The cells expressed genes, as shown in PCR and proteomic data. There have been limited proteomic studies of cartilage, which may be because of the difficulty in determining the amount of protein contribution by the cells relative to the total protein contributed by the ECM [80]. The collagen fibers of the ECM were maintained during the process. Characterization of the scaffold showed that thirty-nine collagen alpha chains were detected in the cartilage prior to decellularization by mass spectrometry. After the decellularization process, nine of these were decreased below the limit of detection. While most of the collagens were maintained throughout the decellularization process, nine were depleted to the extent that they were no longer detectable by mass spectrometry.

SEM was used to visualize the surface topology of the decellularized scaffold [19,23,75,81]. The scaffold was shown to be less smooth and displayed exposed collagenous fibrillar networks after decellularization in SEM and more porous by histology when compared to the original material. SEM images showed cells could attach and proliferate on the surface of the scaffold.

Previous work has not fully analyzed what is left behind after decellularization. Future work should be carried out to better understand the material remaining after decellularization as it is important when evaluating laboratory-generated cartilage for patient-specific biocompatibility. This study is novel because it generates new knowledge by analyzing the protein profile of the decellularized tissue and contributes biomarkers other than well-established COL2A1 and ACAN. It provides an improved approach to monitoring decellularization and insight into acceptable markers for decellularization by looking at the protein profile of markers instead of just one or two proteins. The use of patient-specific MSCs or pre-chondrocytes will advance this line of research. A limitation of this study was that it focused on short-term changes. The scaffolds grown up to eight months showed an increase in cellular adhesion and proliferation. Future experiments in cell culture should extend duration as well as monitor protein synthesis and accumulation over time by proteomics, since protein expression does not always follow RNA expression. In this case, we will identify newly synthesized human proteins based on the number of unique peptides for human proteins for quantification. Additionally, it will be important to test biocompatibility. Another limitation may be the dense nature of native cartilage which restricts cell migration into the matrix. To address this limitation, future studies will include introduction of pores to allow penetration of cells into the center of the scaffold. An alternative approach that will be considered for future studies is devitalized cartilage to deliver higher concentrations of endogenous growth factors that may aid in cellular differentiation. In our procedure, soluble growth factors were removed due to the harsh conditions used.

Overall, this novel research shows promise that laboratory-generated cartilage may be a future alternative treatment option for individuals suffering from OA. Cartilage discs can be made to fit specific cartilage lesions. This approach aims to restore the patient’s natural anatomy and prevent the need of a joint replacement. Using decellularization to create biomaterials can generate biocompatible scaffolds. Patient-specific chondrocytes may promote formation of a tissue that has superior compatibility for replacement or healing of damaged tissue.

## 4. Materials and Methods

### 4.1. Materials

Four pig ears were acquired from Wakefield Meats in Melba, Idaho. The pigs were estimated to be one year of age and of adult size.

### 4.2. Methods

The pig ears were shaved to remove hair, and a scalpel was used to remove remaining skin without damaging the underlying cartilage layer. Samples were placed in a 0.5 M NaOH bath overnight.

Tissue was transferred to a 1.0 M NaOH solution for three hours followed by transfer to a 70% ethanol solution and incubated at 40 °C with heating for three hours.

Pig ear cartilage was converted into 8 mm circular discs using a tissue punch. Twenty 8 mm punches were frozen to be used for characterization of the cartilage-derived scaffold before decellularization.

#### 4.2.1. Decellularization

Three hundred and twenty 8 mm cartilage discs with a thickness of 1.08 mm underwent a decellularization cycle. Then, 1 M guanidine hydrochloride and 0.05 M sodium acetate were used to incubate with agitation at 4 °C for 96 h as described in Schwarz et al. 2012 [75]. Samples were subjected to three freeze–thaw cycles in 1% PBS [19,23,76,77,78,79]. Samples were washed in 10 mM Tris hydroxymethyl aminomethane (Tris)–HCl, 2 mM ethylenediaminetetraacetic acid (EDTA), 5 mM MgCl_2_, 100 mM dithiothreitol (DTT), 1% SDS, and 1% Triton-X100, pH of 8.0, for 39 h with agitation at 22 °C, as described [19,23,74,76,81]. To remove HA and proteoglycans, the cartilage discs were incubated in PBS with 21 U/mL of hyaluronidase at 37 °C for 24 h as described in Luo et al. 2016 [23]. Subsequently, the samples were treated with DNase and RNase for 24 h at 37 °C to degrade DNA and RNA [19,23].

Residual cells were identified based on H&E staining. SEM was performed on the nondecellularized and decellularized tissues at this intermediate step. A second series of washes was carried out to remove the residual cells. Samples underwent another freeze–thaw cycle in DI water and 2% SDS with agitation. Samples were treated with DNase for 72 h at 37 °C with agitation, and samples were analyzed by histology, Hoechst staining, and SEM.

A third series of decellularization washing cycles was carried out followed by lyophilization. DNA removal was confirmed by extraction and purification of total DNA using DNeasy Kit for purification of total DNA from animal tissue (Qiagen).

#### 4.2.2. Histology

The cartilage samples were fixed in 4% paraformaldehyde (PFA) for 1 h and then stored in 35% ethanol at 4 °C and then dehydrated, cleared in Histoclear, and embedded in paraffin. The tissue block was sectioned to achieve both transverse and cross-section configuration on the slide and stained with H&E and Hoechst stain. Hoechst stain (1 µg/mL) was placed on the sample for 5 min, then rinsed 3 times with PBS. The samples were then imaged on a Zeiss Confocal LSM 510 Meta microscope.

#### 4.2.3. SEM Preparation and Imaging

Cartilage discs were fixed in 2.5% glutaraldehyde and 1% osmium tetroxide in Nanopure water. After fixation, samples underwent dehydration with 50%, 70%, 90%, and 100% ethanol. Critical point drying was performed for 10 cycles at 5 °C then heated to 35 °C. Samples were positioned onto an aluminum stub and sealed under vacuum. Gold sputtering was performed at 0.15 megabar (mbar) and 10 milliamp (mA) for 15 cycles of 60 s sputtering and 60 s of rest. Prepped samples were examined at an accelerating voltage of 15 kilovolts (kV) using the secondary electron detector.

#### 4.2.4. PCR

RNA from cells seeded on cartilage scaffold or from cells grown on conventional tissue culture plastic was extracted using TRIzol. Complementary DNA (cDNA) synthesis was carried out using the RT2 First Strand Kit (Qiagen) followed by qRT-PCR in a 96-well plate using a Roche LightCycler96^®^. The samples underwent one cycle for 10 min at 95 °C and then 45 cycles of 15 s at 95 °C and 1 min of 60 °C. Analyzed genes included extracellular matrix proteins, matrix remodeling enzymes, and cell adhesion molecules. PCR primers were purchased from Qiagen (Cat. no. 330231 PAHS-013ZA). Relative gene expression levels, mean of three replicates plus/minus standard deviation, were expressed with respect to housekeeping genes determined empirically for this study.

#### 4.2.5. Protein Concentration Determination

The total protein concentration of the homogenate for all samples was determined using Pierce^TM^ BCA (Bicinchoninic Acid) Protein Assay Kit, Thermo Scientific^®^.

#### 4.2.6. Mass Spectrometry and Proteomics

Proteins from nondecellularized, decellularized, and recellularized scaffolds were homogenized and extracted using RIPA buffer (Millipore, Billerica, MA) Twenty micrograms of total protein from each sample was digested with Trypsin/Lys C mix (Promega, Madison, WI) following the manufacturer’s instruction. Resulting peptide mixtures were chromatographically separated on a reverse-phase C18 column (10 cm × 75 µm, 3 µm, 120 Å) and analyzed on a Velos Pro Dual-Pressure Linear Ion Trap mass spectrometer (Thermo Fisher Scientific).

Peptide spectral matching and porcine and human protein identification were achieved by database search using Sequest HT algorithms in a Proteome Discoverer 2.2 (Thermo Fisher Scientific). Raw spectrum data were searched against the UniProtKB/Swiss-Prot and TrEMBL porcine protein databases and Swiss-Prot human protein database (25 May 2019). The main search parameters included trypsin, maximum missed cleavage site of two, precursor mass tolerance of 1.5 Da, fragment mass tolerance of 0.8 Da, and variable modification of oxidation/hydroxylation of methionine, proline, and lysine (+15.995 Da). A decoy database search was performed to calculate a false discovery rate (FDR). Proteins containing one or more peptides with FDR ≤ 0.05 were considered positively identified and reported. For all proteins, the total number of peptide spectral matches (PSMs) reported by the Protein Discoverer 2.2 was used for quantification. The mass spectrometry analysis used three samples at each condition and time point.

#### 4.2.7. Recellularization of Decellularized Scaffold

Final decellularized cartilage scaffold was sterilized in 70% ethanol and rehydrated in 10% PBS for 24 h at 4 °C followed by incubation in Dulbecco’s modified Eagle’s medium (DMEM) with 10% fetal bovine serum (FBS) and 1% penicillin–streptomycin).

Twenty-four-well plates were prepared with 300 µL of agarose gel in the bottom of each well and sterilized cartilage scaffold discs were seeded with 500,000 C28/I2 cells each. Characterization took place at seven days for PCR analysis and one week and 8 months for SEM analysis.

#### 4.2.8. Statistical Analysis

Selection of housekeeping genes for qRT-PCR was based on pairwise analysis of variance for differences between cycle threshold values for five candidate housekeeping genes from 15 samples within this study. Relative expression of genes of interest was analyzed relative to average values for GAPDH and HPRT1 and expressed as mean plus/minus standard deviation. Log-transformed gene expression data were subject to a paired T-test to determine if the differences in mean values for relative gene expression were statistically significant with *p* < 0.05.

## Figures and Tables

**Table 1:** Proteomic analysis of proteins removed by decellularization.

Nuclear Proteins Depleted by Decellularization Process	% Depletion	Gene Symbol
Aprataxin	100	APTX
BRCA1-A complex subunit RAP80	100	UIMC1
Doublesex- and mab-3-related transcription factor 1	100	DMRT1
Histone H3.3	100	H3F3A
Interferon-induced GTP-binding protein Mx1	100	MX1
Interferon-stimulated gene 20 kDa protein	100	ISG20
Iron-responsive element-binding protein 2	100	IREB2
Myocardin	100	MYOCD
Nuclear factor of activated T-cells, cytoplasmic 1	100	NFATC1
Nuclear receptor subfamily 0 group B member 1	100	NR0B1
Polypyrimidine tract-binding protein 1	100	PTBP1
POU domain, class 5, transcription factor 1	100	POU5F1
Sorbin and SH3 domain-containing protein 2	100	SORBS2
SRSF protein kinase 3	100	SRPK3
Sterol regulatory element-binding protein 1	100	SREBF1
Histone H4	96	Histone H4
Signal transducer and activator of transcription 5A	89	STAT5A
Hepatocyte nuclear factor 1-beta	85	HNF1B
Pre-mRNA-splicing factor ATP-dependent RNA helicase DHX16	85	DHX16
V(D)J recombination-activating protein 1	83	RAG1

**Table 2:** Proteomic analysis of Golgi protein depletion by decellularization.

Golgi Proteins	% Depletion	Gene Symbol
UDP-GalNAc:beta-1,3-N-acetylgalactosaminyltransferase 1	100	B3GALNT1
Mannosyl-oligosaccharide 1,2-alpha-mannosidase IA	100	MAN1A1
Galactoside 2-alpha-l-fucosyltransferase 2	100	FUT2
Alpha-1,3-mannosyl-glycoprotein 4-beta-N-acetylglucosaminyltransferase C	100	MGAT4C
Lactosylceramide 1,3-N-acetyl-beta-d-glucosaminyltransferase	66	B3GNT5

**Table 3:** Mitochondrial proteins depleted during decellularization.

Mitochondrial Proteins	% Depletion	Gene Symbol
Aconitate hydratase, mitochondrial	100	ACO2
A-kinase anchor protein 10, mitochondrial	100	AKAP10
Aspartate aminotransferase, mitochondrial	100	GOT2
Carnitine O-palmitoyltransferase 1, muscle isoform	100	CPT1B
Cholesterol side-chain cleavage enzyme, mitochondrial	100	CYP11A1
Cysteine protease ATG4D	100	ATG4D
Cytochrome b-245 heavy chain	100	CYBB
Cytochrome c oxidase copper chaperone	100	COX17
Cytochrome P450 11B1, mitochondrial	100	CYP11B1
Glycerol-3-phosphate acyltransferase 1, mitochondrial	100	GPAM
Glycine amidinotransferase, mitochondrial	100	GATM
Methylmalonyl-CoA mutase, mitochondrial	100	MUT
Mitochondrial Rho GTPase 2	100	RHOT2
Mitochondrial uncoupling protein 2	100	UCP2
Mitochondrial uncoupling protein 3	100	UCP3
NADH-ubiquinone oxidoreductase chain 5	100	MT-ND5
Succinate dehydrogenase [ubiquinone] flavoprotein subunit, mitochondrial	100	SDHA
Succinate-CoA ligase [ADP/GDP-forming] subunit alpha, mitochondrial	100	SUCLG1
Valine-tRNA ligase, mitochondrial	100	VARS2
Amine oxidase [flavin-containing] B	91	MAOB
Mitochondria-eating protein	91	SPATA18
Nicotinamide phosphoribosyltransferase	91	NAMPT
Hexokinase-2 OS=*Sus scrofa*	87	HK2
Kynurenine 3-monooxygenase	85	KMO
NADP-dependent malic enzyme	83	ME1
Cytochrome P450 3A29	78	CYP3A29
Glyceraldehyde-3-phosphate dehydrogenase	66	GAPDH
Hydroxymethylglutaryl-CoA synthase, mitochondrial	66	HMGCS2
Creatine kinase U-type, mitochondrial	55	CKMT1

**Table 4:** Noncollagenous extracellular matrix proteins after decellularization.

Extracellular Matrix Noncollagenous Proteins	% Depleted	Gene Symbol
Fibromodulin	100	FMOD
Dystroglycan	100	DAG1
Fibrillin-1	100	FBN1
Aggrecan core protein	100	ACAN
Decorin	100	DCN
Lactadherin	98	MFGE8
Hyaluronan and proteoglycan link protein 1	90	HAPLN1
Tenascin	70	TNC
Biglycan	57	BGN

**Table 5:** Genes unique to scaffold compared to growth on tissue culture plastic.

Gene Symbol	Name	Function	Reference
CLEC3B	C-type lectin domain family 3, member B	Encodes tetranectin. Cellular response to transforming growth factor stimulus.	Steinberg 2017 [24] Valdes 2011 [25] Karlsson 2010 [26] Mazzoni 2020 [27]
COL12A1	Collagen, type XII, alpha 1	Encodes the alpha chain of type XII collagen. Modifies the interactions between collagen fibrils and the surrounding matrix. A component of cartilage ECM.	Johnson 2015 [28] Zeggini 2012 [29] Manon-Jensen 2016 [30] Luo 2017 [31] Agarwal 2012 [32]
COL15A1	Collagen, type XV, alpha 1	Encodes the alpha chain of type XV collagen. Strongest expression in basement membrane zones; may function to adhere basement membranes to underlying connective tissue.	Karlsson 2010 [26] Zhou 2010 [33] Valdes 2011 [25]
CTGF	Connective tissue growth factor	Modulates signaling pathways leading to cell adhesion and migration, along with ECM deposition and remodeling, which together lead to tissue remodeling.	Tang 2018 [34] Ivkovic 2003 [35] Shi-Wen 2008 [36] Lipson 2012 [37]
ECM1	Extracellular matrix protein 1	Inhibits chondrocyte hypertrophy, matrix mineralization, and endochondral bone formation.	Kong 2016 [38] Mongiat 2003 [39] Frahs 2019 [40] Kong 2010 [41]
ICAM1	Intercellular adhesion molecule 1	Encodes cell surface glycoprotein.	Yatabe 2009 [42] Gromova 2018 [43] Rangkasenee 2013 [44]
ITGA3	Integrin, alpha 3 (antigen CD49C, alpha 3 subunit of VLA-3 receptor)	Involved in cell adhesion and collagen binding.	Zhang 2019 [45]
ITGA4	Integrin, alpha 4 (antigen CD49D, alpha 4 subunit of VLA-4 receptor)	Functions in cell surface adhesion and signaling, ECM receptor interaction.	Djouad 2007 [46] Weeks 2012 [47] Zhu 2017 [48]
ITGA6	Integrin, alpha 6	Functions in cell surface adhesion and signaling.	Tu 2020 [49] LaPointe 2013 [50]
LAMA1	Laminin, alpha 1	Major component of the basement membrane. Associated with cell adhesion, differentiation, migration, and signaling.	Zhang 2019 [45] Wang 2019 [51] Soki 2018 [52] Adapala 2016 [53] Grogan 2013 [54] Mann 2019 [55]
MMP10	Matrix metallopeptidase 10 (stromelysin 2)	Involved in the breakdown of extracellular matrix in normal physiological processes, such as tissue remodeling.	Dehne 2010 [56] Gohring 2010 [57]
MMP12	Matrix metallopeptidase 12	Involved in the breakdown of extracellular matrix in normal physiological processes, such as tissue remodeling.	Dehne 2010 [56] Lv 2016 [58]
MMP9	Matrix metallopeptidase 9 (gelatinase B, 92 kDa gelatinase, 92 kDa type IV collagenase)	Involved in the breakdown of extracellular matrix in normal physiological processes, such as tissue remodeling.	Challa 2010 [59] Yang 2015 [60] Miao 2004 [61]
SELP	Selectin P (granule membrane protein 140 kDa, antigen CD62)	This protein redistributes to the plasma membrane during platelet activation and degranulation.	Weeks 2012 [47] Gari 2016 [62] Rouillard 2016 [63] Bonn 2010 [64]
THBS3	Thrombospondin 3	Mediates cell-to-cell and cell-to-matrix interactions. Found in developing cartilage.	Vos 1992 [65] Adolph 1995 [66] Djouad 2007 [46] Posey 2008 [67] Hankenson 2005 [68]
VCAN	Versican	Major component of the ECM; involved in cell adhesion and proliferation during chondrogenesis.	Kamiya 2006 [69] Choocheep 2010 [70] Sztrolovics 2002 [71]
VTN	Vitronectin	ECM markers that promote cell adhesion and spreading.	Luo 2017 [72] Vieira 2015 [73] Pei 2013 [74]

## Data Availability

The datasets used and/or analyzed during the current study are available from the corresponding author upon request.

## Appendix A

**Table A1:** Endoplasmic reticulum proteins depleted during decellularization.

Endoplasmic Reticulum	% Depletion	Gene Symbol
40S ribosomal protein S13	100	RPS13
40S ribosomal protein S3	100	RPS3
5-beta-cholestane-3-alpha,7-alpha-diol 12-alpha-hydroxylase	100	CYP8B1
60S ribosomal protein L14	100	RPL14
60S ribosomal protein L6	100	RPL6
Beta-crystallin B1	100	CRYBB1
Dolichyl-diphosphooligosaccharide--protein glycosyltransferase subunit 2	100	RPN2
Endoplasmic reticulum chaperone BiP	100	HSPA5
Glutamate carboxypeptidase 2	100	FOLH1
Heat shock 70 kDa protein 1A	100	HSPA1A
Heat shock 70 kDa protein 1B	100	HSPA1B
Heat shock 70 kDa protein 1-like	100	HSPA1L
Heme oxygenase 1	100	HMOX1
Neutral alpha-glucosidase AB	100	GANAB
Sarcoplasmic/endoplasmic reticulum calcium ATPase 2	100	ATP2A2
Transitional endoplasmic reticulum ATPase	100	VCP
Microsomal triglyceride transfer protein large subunit	85	MTTP
Dolichyl-diphosphooligosaccharide--protein glycosyltransferase subunit 1	78	RPN1
Dual oxidase 1	78	DUOX1
Heat shock protein HSP 90-alpha	78	HSP90AA1

**Table A2:** Membrane proteins depleted during decellularization.

Membrane Proteins	% Depletion	Gene Symbol
ADP-ribosylation factor 6	100	ARF6
Arachidonate 15-lipoxygenase	100	ALOX15
Calpain-1 catalytic subunit	100	CAPN1
C-X-C chemokine receptor type 4	100	CXCR4
Cysteinyl leukotriene receptor 2	100	CYSLTR2
Desmoglein-1	100	DSG1
Endothelin-1 receptor	100	EDNRA
Gap junction alpha-1 protein	100	GJA1
Geranylgeranyl transferase type-2 subunit alpha	100	RABGGTA
Glutathione hydrolase 1 proenzyme	100	GGT1
Growth hormone receptor	100	GHR
Guanine nucleotide-binding protein G(q) subunit alpha	100	GNAQ
Integrin beta-1	100	ITGB1
Interferon alpha/beta receptor 1	100	IFNAR1
Interleukin-4 receptor subunit alpha	100	IL4R
Interleukin-6 receptor subunit alpha	100	IL6R
Low-density lipoprotein receptor	100	LDLR
Mast/stem cell growth factor receptor Kit	100	KIT
Metalloreductase STEAP1	100	STEAP1
NT-3 growth factor receptor	100	NTRK3
Parathyroid hormone/parathyroid hormone-related peptide receptor	100	PTH1R
PDZ domain-containing protein 11	100	PDZD11
Plasma membrane calcium-transporting ATPase 1	100	ATP2B1
Platelet endothelial cell adhesion molecule	100	PECAM1
Protocadherin-11 X-linked	100	PCDH11X
Receptor activity-modifying protein 1	100	RAMP1
S-Arrestin	100	SAG
Sialoadhesin	100	SIGLEC1
SLA class II histocompatibility antigen, DQ haplotype C alpha chain	100	SLA-DQCA
SLA class II histocompatibility antigen, DQ haplotype C beta chain	100	SLA-DQCB
SLA class II histocompatibility antigen, DQ haplotype D beta chain	100	SLA-DQDB
Small conductance calcium-activated potassium channel protein 3	100	KCNN3
Sodium/glucose cotransporter 1	100	SLC5A1
Solute carrier family 22 member 6	100	SLC22A6
Solute carrier family 22 member 7	100	SLC22A7
Thyroid peroxidase	100	TPO
Toll-like receptor 9	100	TLR9
Transforming growth factor beta receptor type 3	100	TGFBR3
Beta-1 adrenergic receptor	94	ADRB1
Zonadhesin	94	ZAN
Glutathione S-transferase alpha M14	94	GSTAM14
Activin receptor type-2B	93	ACVR2B
Solute carrier family 22 member 1	91	SLC22A1
Low-density lipoprotein receptor-related protein 2	89	LRP2
Orexin receptor type 2	89	HCRTR2
Glutamate decarboxylase 2	89	GAD2
Ectonucleotide pyrophosphatase/phosphodiesterase family member 6	85	ENPP6
Tyrosine-protein kinase SYK	85	SYK
Hormone-sensitive lipase	85	LIPE
V-type proton ATPase catalytic subunit A	85	ATP6V1A
Potassium-transporting ATPase alpha chain 1	82	ATP4A
Electrogenic sodium bicarbonate cotransporter 1	81	SLC4A4
Alpha-2A adrenergic receptor	78	ADRA2A
Calcium-activated chloride channel regulator 1	78	CLCA1
Gastrin/cholecystokinin type B receptor	78	CCKBR
Hepatocyte growth factor receptor	78	MET
Leptin receptor	78	LEPR
Extracellular calcium-sensing receptor	70	CASR
Scavenger receptor class B member 1	70	SCARB1
ATP-binding cassette sub-family G member 2	66	ABCG2
Major facilitator superfamily domain-containing protein 6	66	MFSD6
H(+)/Cl(−) exchange transporter 5	63	CLCN5
Prolactin receptor	55	PRLR
Beta-3 adrenergic receptor	55	ADRB3
Lutropin-choriogonadotropic hormone receptor	55	LHCGR

**Table A3:** Cytosolic proteins depleted during decellularization.

Cytosolic Proteins	% Depletion	Gene Symbol
1-acylglycerol-3-phosphate O-acyltransferase ABHD5	100	ABHD5
4-hydroxyphenylpyruvate dioxygenase	100	HPD
Actin, cytoplasmic 1	100	ACTB
Alcohol dehydrogenase [NADP(+)]	100	AKR1A1
Antileukoproteinase	100	SLPI
ATP-dependent 6-phosphofructokinase, muscle type	100	PFKM
Autophagy protein 5	100	ATG5
Bifunctional epoxide hydrolase 2	100	EPHX2
Biogenesis of lysosome-related organelles complex 1 subunit 5	100	BLOC1S5
Calponin-1 OS = *Sus scrofa*	100	CNN1
Calponin-2	100	CNN2
Cas scaffolding protein family member 4	100	CASS4
Coatomer subunit beta	100	COPB1
Diacylglycerol kinase alpha	100	DGKA
Dihydropyrimidine dehydrogenase [NADP(+)]	100	DPYD
FAST kinase domain-containing protein 4	100	TBRG4
Gastrotropin	100	FABP6
Growth factor receptor-bound protein 10	100	Grb10
Integrin beta-1-binding protein 2	100	ITGB1BP2
L-dopachrome tautomerase	100	DCT
L-lactate dehydrogenase A chain	100	LDHA
Myosin light chain 4	100	MYL4
Myosin-1	100	MYH1
Myosin-2	100	MYH2
Nucleoside diphosphate kinase B	100	NME2
Perilipin-3	100	PLIN3
Phosphatidylinositol 4,5-bisphosphate 3-kinase catalytic subunit gamma isoform	100	PIK3CG
Serine/threonine-protein phosphatase 2A 65 kDa regulatory subunit A beta isoform	100	PPP2R1B
Suppressor of cytokine signaling 2	100	SOCS2
Thimet oligopeptidase	100	THOP1
Triosephosphate isomerase	100	TPI1
Tubulin alpha-1A chain	100	TUBA1A
Tubulin beta chain	100	TUBB
Vinculin	100	VCL
Serine/threonine-protein kinase WNK1	95	WNK1
L-lactate dehydrogenase B chain	94	LDHB
UTP-glucose-1-phosphate uridylyltransferase	92	UGP2
Eukaryotic initiation factor 4A-III	89	EIF4A3
Triokinase/FMN cyclase	85	TKFC
Acylphosphatase-1	83	ACYP1
Glycine N-methyltransferase	78	GNMT
N-acetylneuraminate lyase	78	NPL
Phosphatidylinositol 3-kinase catalytic subunit type 3	78	PIK3C3
Serine/threonine-protein phosphatase 1 regulatory subunit 10	64	PPP1R10
